# Local structure of solid and liquid gold probed by reverse Monte Carlo analysis of X-ray absorption data

**DOI:** 10.1107/S1600577524011706

**Published:** 2025-02-04

**Authors:** Nodoka Hara, Fabio Iesari, Toshihiro Okajima, Andrea Di Cicco

**Affiliations:** a Physics Division, School of Science and Technologies University of Camerino Via Madonna delle Carceri 9 62032Camerino MC Italy; b European XFEL 22869 Schenefeld Germany; c Aichi Synchrotron Radiation Center, Seto, Aichi489-0965, Japan; University of Essex, United Kingdom

**Keywords:** relativistic corrections, *GNXAS* analysis package, XAFS, reverse Monte Carlo

## Abstract

The increase in computational capabilities of modern computers has allowed us to develop new accurate methods for EXAFS (extended X-ray absorption fine structure) data-analysis. In particular, the *RMC-GNXAS* package provides models of the three-dimensional structure applying the reverse Monte Carlo (RMC) method to EXAFS of condensed or molecular systems. Simulations of the EXAFS signals within *RMC-GNXAS* are based on accurate multiple-scattering (MS) simulations accounting for relativistic effects. The method is implemented to include MS signals related to pair and triplet distributions, using different data sets and long-range constraints provided by complementary techniques (diffraction). In this work we have validated this approach studying the local structure of solid and liquid Au, providing also a benchmark for analysis of functional materials based on heavy noble atoms such as Au. An accurate reconstruction of the pair and triplet distribution functions in solid and liquid Au was obtained and compared with literature data. We show that the inclusion of the contribution of MS signals related to three-body configurations is especially important in solid Au at low temperatures. In liquid Au, we obtained the pair distribution function and analyzed the bond–angle distribution. We applied common-neighbor analysis to identify the local symmetry of the atomic configurations. Liquid Au is found to follow the trend of other close-packing liquids for which the dominant structures are distorted or defective icosahedral configurations.

## Abstract 2

This study demonstrates the validation of the *RMC-GNXAS* method including two- and three-body EXAFS signals for the local structure analysis of both solid and liquid Au as a benchmark for the study of heavy noble metal systems.

## 1. Introduction

Gold is a noble metal with atomic number *Z* = 79, often regarded as a transition metal due to the *sd* character of the conduction band. Its crystal configuration at ambient conditions is face-centered-cubic (f.c.c.) with 12 nearest neighbors. Gold is widely used as a reference material in basic and applied sciences and is presently used in applied research as a functional material in several forms for a variety of applications including catalysts (Haruta *et al.*, 1987 ▸; Tibiletti *et al.*, 2005 ▸; Hashmi *et al.*, 2010 ▸), nano-clusters (Häkkinen, 2008 ▸), nano-particles (Comaschi *et al.*, 2008 ▸) and also in shock compression experiments to generate high pressure environments for phase transition studies (Briggs *et al.*, 2019 ▸; Sharma *et al.*, 2019 ▸; Wu *et al.*, 2020 ▸).

Knowledge of the local structure of gold systems is a pre-requisite for understanding possible functional properties of ordered or disordered materials based on Au. Among other techniques, X-ray absorption spectroscopy (XAS) is a powerful tool for investigating the local structure of ordered, disordered or ill-ordered materials through the study of the so-called X-ray absorption fine structure (XAFS or EXAFS). Modern XAFS data analysis is based on the refinement of experimental data using accurate multiple-scattering (MS) calculations, and several simulation/fitting codes for XAFS have been developed for this purpose [see, for example, Rehr & Albers (2000 ▸), Filipponi *et al.* (1995 ▸) and Filipponi & Di Cicco (1995 ▸)]. Standard EXAFS analysis is based on the so-called peak-fitting technique, where the structural parameters are related to the individual distance distributions of the neighboring shells. Treatment of the configurational average of MS signals can be considerably complicated but it has been shown (Filipponi *et al.*, 1995 ▸) that *n*-body expansion is rapidly convergent so lower-order terms associated with pairs [two-body signals: γ^(2)^] and triplets [three body signals: γ^(3)^] of atoms are usually sufficient. The peak-fitting approach over the *g*(*r*) distributions works fine for crystals and may be extended also to moderate disordered systems (Bunker, 1983 ▸; Di Cicco *et al.*, 2000 ▸; a Beccara *et al.*, 2003 ▸) and simple liquids (Filipponi, 2001 ▸; Trapananti & Di Cicco, 2004 ▸) but it shows severe limitations in describing the local structure of strongly anharmonic systems, nanocrystalline, highly disordered materials and complex liquids.

Model-independent data-analysis schemes, comparing directly simulations of tridimensional model structures with EXAFS experimental data, have also been developed in the last decades. One of the most promising schemes is based on the application of the reverse Monte Carlo (RMC) method (McGreevy & Pusztai, 1988 ▸), which has been extended to include EXAFS refinements in various ways (Gurman & McGreevy, 1990 ▸; Winterer, 2000 ▸; Di Cicco *et al.*, 2003 ▸; Di Cicco & Trapananti, 2005 ▸; Timoshenko *et al.*, 2012 ▸; Di Cicco & Iesari, 2022 ▸). Of course, these complex data-analysis schemes require larger interpretation efforts and computer time, but recent advances in software and computing have made RMC analysis feasible on more regular computing resources. An important feature of the RMC method is the possibility of refining multiple sets of data; so, for example, EXAFS can be conveniently associated with diffraction data. In this way, complementary information on short- and medium-range ordering can be obtained on any material of interest. In particular, *RMC-GNXAS* (Di Cicco & Trapananti, 2005 ▸; Di Cicco & Iesari, 2022 ▸) is an RMC structure analysis method that provides refinements of EXAFS data by accurate MS simulations taking into account pair distribution [*g*_2_(*r*)] models obtained by other techniques (diffraction or computer simulations).

The aim of this work is to show that reliable information about the local structure of elemental Au in the solid and liquid phase can be obtained by the application of the *RMC-GNXAS* method. This can provide a benchmark for applications on more complex functional materials based on Au. The application to Au is particularly challenging as it needs relativistic treatment of the MS signals and the inclusion of three-body contributions related to the f.c.c. structure. In fact, in previous work (Tchoudinov *et al.*, 2022 ▸; Hara *et al.*, 2023 ▸), we have already applied the standard ‘peak-fitting’ scheme using *GNXAS* to determine the first neighbor distribution in solid and liquid phases including relativistic corrections (Hara *et al.*, 2021 ▸), which is necessary for the analysis of systems containing heavy elements (Tchoudinov *et al.*, 2022 ▸), confirming also the presence of three-body MS signals due to the collinear configurations in the f.c.c. structure. Thanks to recent developments (Di Cicco *et al.*, 2003 ▸; Iesari *et al.*, 2025 ▸), three-body contributions can be explicitly included in RMC refinements and reliable structure analysis can be performed also in crystalline solids at low temperatures.

In this paper, we present the three-dimensional structure refinement of crystalline Au at 80 and 300 K, as well as liquid Au at 1400 K [the melting point of Au is 1337 K (Pamato *et al.*, 2018 ▸)], using Au *L*_3_-edge XAFS with the RMC method as a benchmark for heavy element systems, while taking the relativistic effect of phase shifts into account. This paper consists of the following: in Section 2 we discuss EXAFS data analysis using the RMC method for solid and liquid Au phases; Section 3 is devoted to the conclusions.

## 2. Structural refinement by XAS with RMC

Au EXAFS *L*_3_-edge measurements have been collected at 80 and 300 K for the solid phase and 1400 K for the liquid phase. Details of this experiment are described by Hara *et al.* (2023 ▸). *RMC-GNXAS* structural refinements have been performed as usual (Di Cicco & Trapananti, 2005 ▸; Di Cicco & Iesari, 2022 ▸), starting from an initial atomic configuration representative of the system under consideration.

After constructing the initial configuration, the starting EXAFS signals and radial distribution functions are calculated. The residual function for *RMC-GNXAS* refinement of this monatomic system is defined as
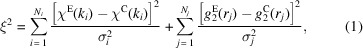
where χ^E^(*k*) and χ^C^(*k*) are the experimental and calculated EXAFS signals, respectively, 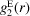 and 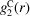 are the model [from X-ray diffraction (XRD) or other techniques] and calculated pair distribution functions, respectively. The χ^C^(*k*) and 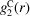 functions are calculated from the atomic coordinates of the box atoms used in the RMC process. The sums in equation (1) are weighted using appropriate noise functions (σ^2^) providing the correct estimate for the statistical χ^2^-like random variable for the iterative RMC refinement (Iesari *et al.*, 2024 ▸). This is done moving each atom randomly in the configuration and the new position is retained or discarded accordingly to the standard Metropolis sampling based on the residual value of equation (1), taking into account possible physical constraints. When all the atom positions have been subject to a possible variation, an RMC move is completed. Typically, after several RMC moves, convergence to an equilibrium structure is reached and several equilibrium configurations can be saved for structural analysis.

An important aspect of this procedure is the combination of EXAFS data and pair distribution functions obtained by other techniques. This allows us to obtain final equilibrium configurations matching short-range and medium-range properties of the system under consideration. Full details on the procedure and input parameters required for an *RMC-GNXAS* simulation are given by Di Cicco & Trapananti (2005 ▸) and Iesari *et al.* (2018 ▸).

As anticipated, the EXAFS signal χ^C^(*k*) is calculated using MS simulations performed within the *n*-body expansion that is usually rapidly convergent so two-body MS signals [γ^(*n*)^, *n* = 2] related to short-range interatomic distances are usually dominant. Depending on the level of disorder and other factors, the EXAFS signal contains mainly two-body and three-body γ^(*n*)^ terms of weaker intensity. In order to keep calculation times within reasonable limits, in previous applications the weak three-body contributions were removed from the experimental signal prior to the *RMC-GNXAS* refinements. In fact, calculating γ^(3)^ signals for each RMC step involves considering triplets of atoms and calculation times become proportional to *N*^3^, where *N* is the number of atoms involved. However, the wider memory and faster processing of modern computers as well as the possibility of using parallel optimization allowed us to develop suitable computational strategies for proper calculation and refinement of three-body terms. The developments in computing strategies are beyond the scope of this paper and will be the subject of a separate work.

Present refinements for solid Au were performed calculating the EXAFS signal χ^C^(*k*) using either only two-body γ^(2)^ MS signals, refinement method (*A*), or both two-body γ^(2)^ and three-body γ^(3)^ signals, refinement method (*B*). The chosen model structure was obviously an f.c.c. cell where the lattice parameter was given by XRD (Pamato *et al.*, 2018 ▸) (*a* = 4.066 and 4.078 Å for 80 K and 300 K, respectively).

RMC refinements using only two-body signals (*A*) have been performed using a box of 7 × 7 × 7 f.c.c. cells corresponding to 1372 Au atoms at a given atomic density. Instead, due to the longer computing times, RMC refinements considering both two-body and three-body signals (*B*) were performed using a box of 4 × 4 × 4 f.c.c. cells (256 atoms). Note that the initial structure, f.c.c., is not a direct constraint. It only provides the correct atomic density in the box. As is evident from Hara *et al.* (2023 ▸), three-body signals γ^(3)^ are not negligible for solid Au, so the experimental signal used for refinements (*A*), χ^E^(*k*), was obtained subtracting the calculated γ^(3)^ contributions found in the cited reference [χ^E^(*k*) − 〈γ^(3)^〉].

Of course, in refinements (*B*) the experimental signal χ^E^(*k*) was just obtained by proper normalization and background removal as presented by Hara *et al.* (2023 ▸).

The initial model structure for liquid Au was again a box of 7 × 7 × 7 f.c.c. cells with a lattice parameter *a* = 4.239 Å corresponding to an atomic density of 0.0525 atoms Å^−3^ at 1423 K, as reported by Waseda (1980 ▸). In this case we verified that the three-body and higher-order signals are negligible so calculations were performed considering only the two-body γ^(2)^ contributions.

All the RMC refinements have been carried out in two successive steps, as in previous works. First, the initial ordered model structure is used in an RMC procedure including only 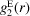 provided by XRD data. Then, this configuration reproducing a model 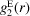 was used as the initial configuration for a full RMC refinement including also EXAFS data. In this work, for both systems, solid and liquid, the RMC refinements have been carried out based on both the measured EXAFS signals χ^E^(*k*) and the pair distribution function 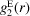 determined by XRD [equation (1)]. We verified that without the constraint provided by 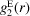 the long-range structure cannot be reproduced correctly, confirming the results reported by Di Cicco & Iesari (2022 ▸).

The calculations of the two-body γ^(2)^ and even three-body γ^(3)^ MS terms were performed using a proper relativistic correction to the scattering *t*-matrix (Tchoudinov *et al.*, 2022 ▸; Hara *et al.*, 2021 ▸).

The χ^E^(*k*) functions are the experimental *L*_3_-edge Au EXAFS spectra reported in our previous work (Hara *et al.*, 2023 ▸). The 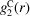 pair distributions are simply obtained by the atomic coordinates at each RMC step. As mentioned, the pair distribution functions 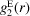 were evaluated at each temperature on the basis of previous works (Hara *et al.*, 2023 ▸) and a correlated Debye model (Sevillano *et al.*, 1979 ▸) for solid and liquid (Waseda, 1980 ▸) Au.

Results and more details on the data-analysis are described in the following subsections.

### 2.1. Solid Au

As anticipated, the first step of the *RMC-GNXAS* analysis is the inclusion of the model pair distribution 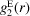 in equation (1) only, without considering the EXAFS signals. In this way, the generated atomic configurations (1000 RMC steps) were fully compatible with the model 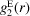 function.

The model pair distributions 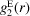 for solid Au were obtained using the *grrec* code (*GNXAS* software) (Di Cicco, 2009 ▸), which generates pair distribution functions based on the input structure parameters with selected peak shape and density. The model pair distributions of solid Au accounted for the known asymmetry of the first-neighbor distribution peak. Therefore a Γ-like distribution (Filipponi *et al.*, 1995 ▸) depending on four parameters (mean distance *R*_1_, mean square relative displacement MSRD or bond variance 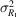, skewness β = *C*_3_/σ^3^ which is related to the third cumulant *C*_3_, and coordination number *N* = 12 in f.c.c.) was used as indicated in Tables 1 ▸ and 2 ▸ (model), based on the value reported by Hara *et al.* (2023 ▸).

For further shells including more distant atoms we used Gaussian peak shapes (β = 0) based on mean distances *R* related to the f.c.c. structure and a correlated Debye model (Sevillano *et al.*, 1979 ▸) for bond variances 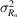 (*n* = 2, 3, 4) using the Debye temperature Θ_D_ = 188 K (Pamato *et al.*, 2018 ▸).

All the parameters related to the model structure are shown in Tables 1 ▸ and 2 ▸ for Au at 80 and 300 K, respectively.

As usual in current *RMC-GNXAS* data-analysis the signal χ^E^(*k*) was obtained by a *GNXAS* pre-analysis and for the calculated EXAFS signal χ^C^(*k*) we kept fixed the non-structural EXAFS parameters 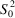 and *E*_0_ to the original values reported by Hara *et al.* (2023 ▸). MS EXAFS signals contributing to the χ^C^(*k*) term were calculated up to a cut-off distance of 6.0 Å using a Gaussian smoothing with 0.3 Å standard deviation to avoid truncation effects [residual high-frequency oscillation in the calculated EXAFS signal, see Filipponi (2001 ▸)]. This includes atoms up to the fourth coordination shell, to take account of collinear configurations showing large three-body contributions due to the strong MS focusing effect.

**Figure 1:**
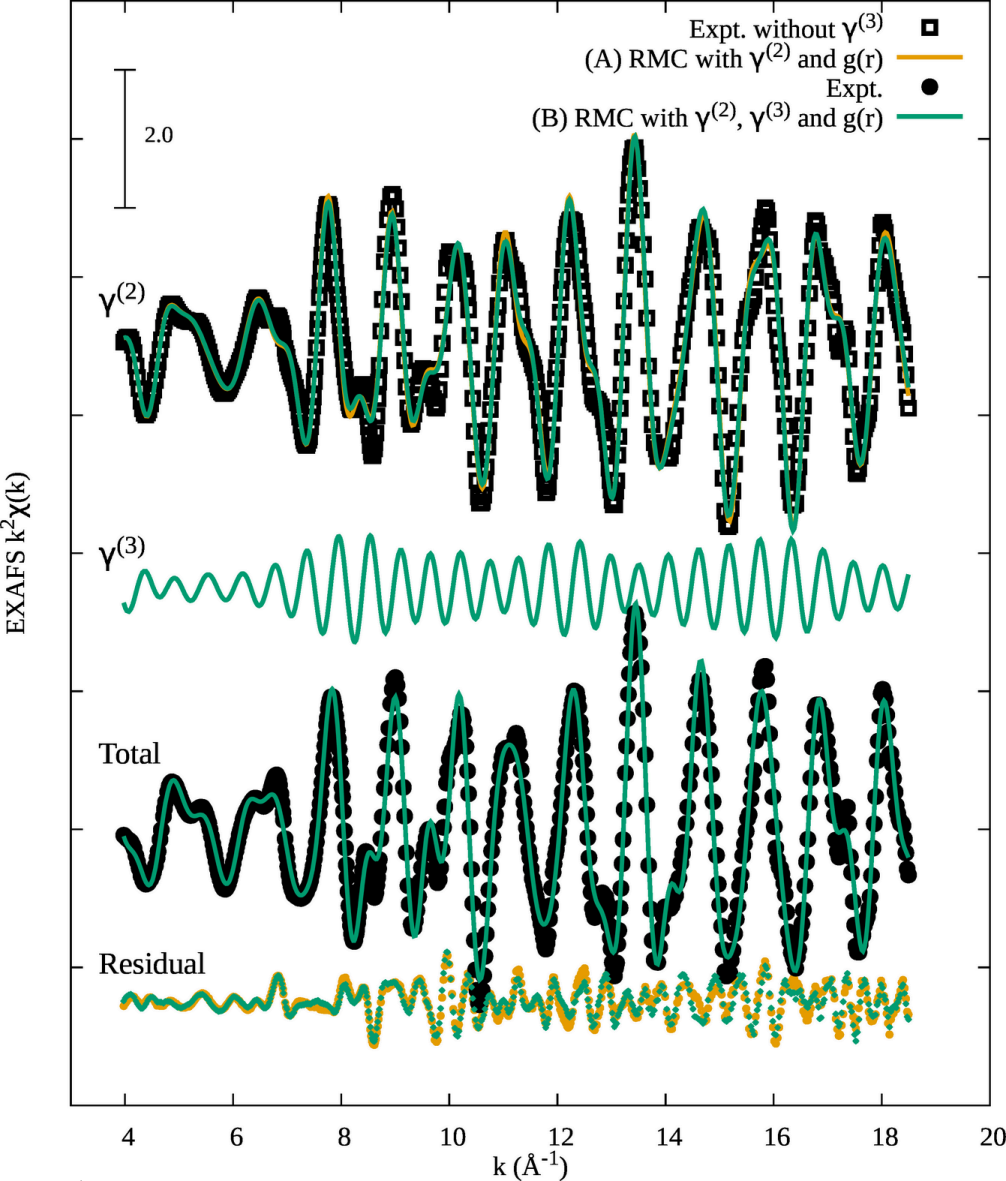
RMC EXAFS refinement results of solid Au at 80 K using the two different strategies (*A*) and (*B*). Experimental EXAFS data (Expt., black circles) are reported also removing the three-body contribution γ^(3)^ (black squares). EXAFS by RMC is indicated by lines and experiment indicated by dots.

As discussed above, we then followed the two different refinement strategies (*A*) and (*B*) for including the EXAFS experimental signal χ^E^(*k*), starting from structures compatible with the model 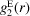 as reported above including γ^(3)^ signals in a different way. As an example, the results of the EXAFS refinements for solid Au at 80 K are shown in Fig. 1 ▸.

The upper curve of Fig. 1 ▸ (black squares) is the experimental EXAFS χ^E^(*k*) spectra without three-body signals χ^E^(*k*) − 〈γ^(3)^〉 as calculated in our previous work using standard peak-fitting analysis (Hara *et al.*, 2023 ▸). This curve is compared with the RMC refinement χ^C^(*k*) (orange curve), obtained in a run of 1000 RMC moves (convergence reached after about 50 RMC moves) including only two-body γ^(2)^ signals. This refinement (*A*) is fast in terms of computer time [∼0.6 s per RMC step, 8 CPU Intel i9, 685 EXAFS data points, 460 *g*(*r*) points] and allows inclusions of a large box of atoms (1372 in this case).

EXAFS RMC analysis including explicitly three-body γ^(3)^ signals (*B*) was also performed on the original EXAFS data χ^E^(*k*) (Fig. 1, lower curve, black circles, Expt.). The initial configuration of this RMC refinement was based on that obtained from method (*A*) and convergence was reached in about 20 RMC moves in a run of 100 RMC moves. In this case, the direct calculation of γ^(3)^ signals (Di Cicco *et al.*, 2003 ▸) for a solid structure also increases computer time, using parallel calculations [∼14500 s per RMC step, 8 CPU Intel i9, 604 EXAFS data points, 460 *g*(*r*) points].

Looking at Fig. 1 ▸ the agreement between experimental EXAFS signals and RMC refinements using both methods (*A*) (orange curves) and (*B*) (green curves) is excellent, as shown by the superimposed curves with experimental data (black dots). The residual (bottom curves) in both cases is similar and contains mainly high-frequency contributions that were not considered in the present model limited to distances within the fourth coordination shell. The total two-body (〈γ^(2)^〉) and three-body (〈γ^(3)^〉) contributions related to the RMC refinements (*A*) and (*B*) are reported in Fig. 1 ▸. It is important to remark that the two-body contributions almost coincide (upper orange and green curves) showing that both methods (*A*) (used in most previous applications) and (*B*) are reliable and that the three-body contribution 〈γ^(3)^〉 (middle curve, green) is weaker but absolutely not negligible for f.c.c. solid systems. This was also found in this solid at different temperatures (300 K), not shown here.

**Figure 2:**
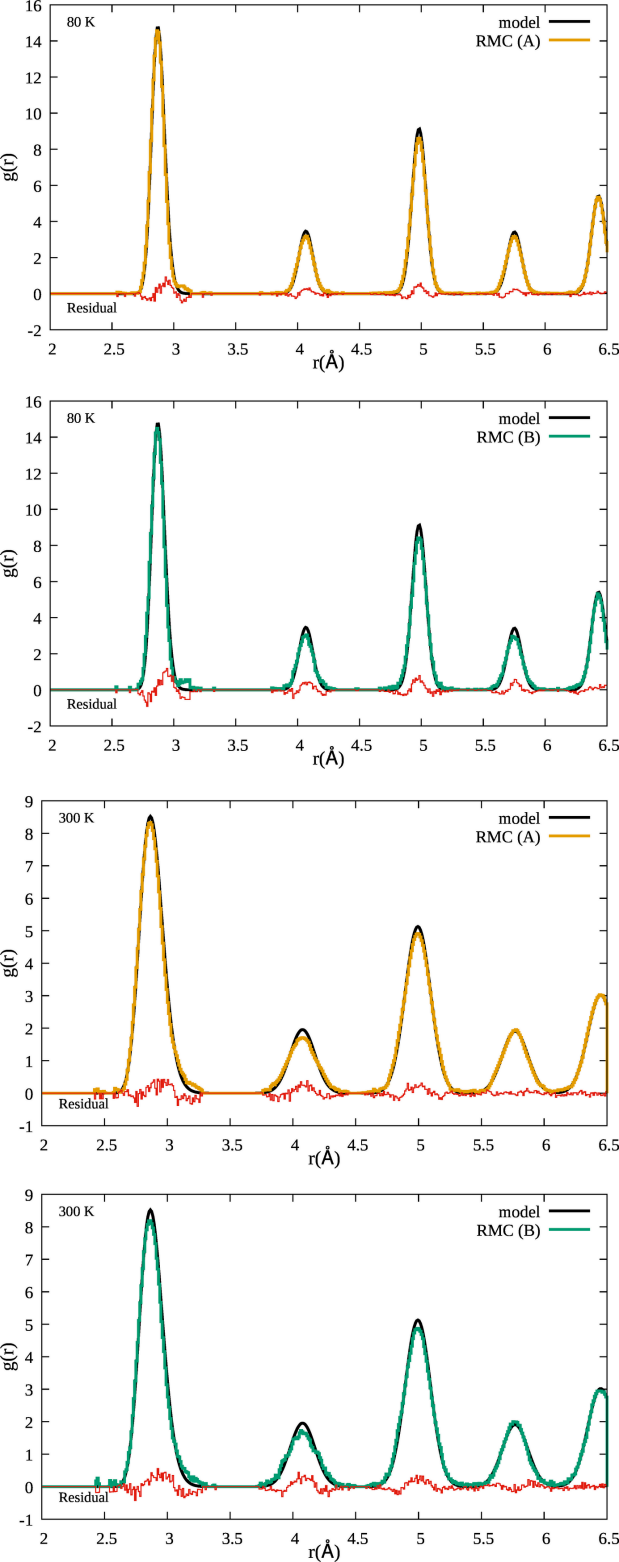
Pair distribution functions *g*(*r*) of solid Au at 80 K (upper panels) and 300 K (lower panels) for RMC refinements (*A*) and (*B*). The pair distributions reconstructed by RMC are compared with those of the models at the two temperatures. An excellent agreement is observed as confirmed by the small residual for each coordination shell.

In Fig. 2 ▸ we show a comparison between pair distribution functions *g*(*r*) used as a model (see Table 2 ▸) and those reconstructed by RMC refinements (*A*) and (*B*) for solid Au at temperatures of 80 K and 300 K. In Fig. 2 ▸ we can see up to five coordination shells within the plot limits [*g*(*r*) is obviously extended at larger distances], the first four included in the actual EXAFS refinement. Broadly speaking, there is an overall nice agreement between the models and the RMC results and obviously the *g*(*r*) peaks are broader at higher temperatures (300 K) as expected due to the increase of the MSRD σ^2^ due to thermal vibrations. The small differences between RMC refinements and model curves for the first four coordination shells are related directly to the EXAFS signals and may be an indication of the known limitations of the Debye model for evaluating σ^2^ values for different interatomic distances and temperatures.

The RMC configurations at equilibrium were also analyzed calculating the cumulants of the distributions related to each peak of the pair distribution functions. The resulting cumulants were used to calculate for each coordination shell the average distances *R*_*n*_, MSRDs 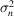, as well as their skewness β and kurtosis τ − 3 = (*C*_4_/σ^4^) − 3, where *C*_4_ is the fourth cumulant (those last two are zero for Gaussian distributions).

The parameters derived from RMC refinements are compared with those of the models for solid Au at 80 and 300 K in Tables 1 ▸ and 2 ▸, respectively. Error values indicated in the two tables are purely statistical and are related to the fluctuations of the parameters for the considered RMC configurations. Of course, they do not include other sources of uncertainties (for example systematic errors in experiments and theory) and are likely to be much smaller than the actual uncertainty. The difference between best-fit values using refinements (*A*) and (*B*) represents a more realistic estimate of the error bars. The values of the parameters are in overall nice agreement with those of the models and, while the first neighbor distribution is confirmed to be asymmetric, the other shells are practically Gaussian functions.

Moreover, RMC configurations were also analyzed to study the triplet distribution considering the three-body configurations up to a cut-off distance *R*_cut_ = 3.4 Å, so including only the first neighbors. The resulting mean angles θ, angular variance 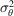, bond–bond ρ_*RR*_ and bond–angle ρ_*R*θ_ correlations are also reported in Tables 1 ▸ and 2 ▸ and represent unconventional structural information obtained by XAFS. Here we defined dimensionless correlation parameters ρ (Filipponi *et al.*, 1995 ▸) in the range (−1, 1) as ρ_*i*,*j*_ = 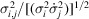. ρ_*R*1*R*1_ is the correlation between first-neighbor bonds (*R*_1_) and ρ_*R*1θ_ is the correlation between bond *R*_1_ and angle θ.

**Figure 3:**
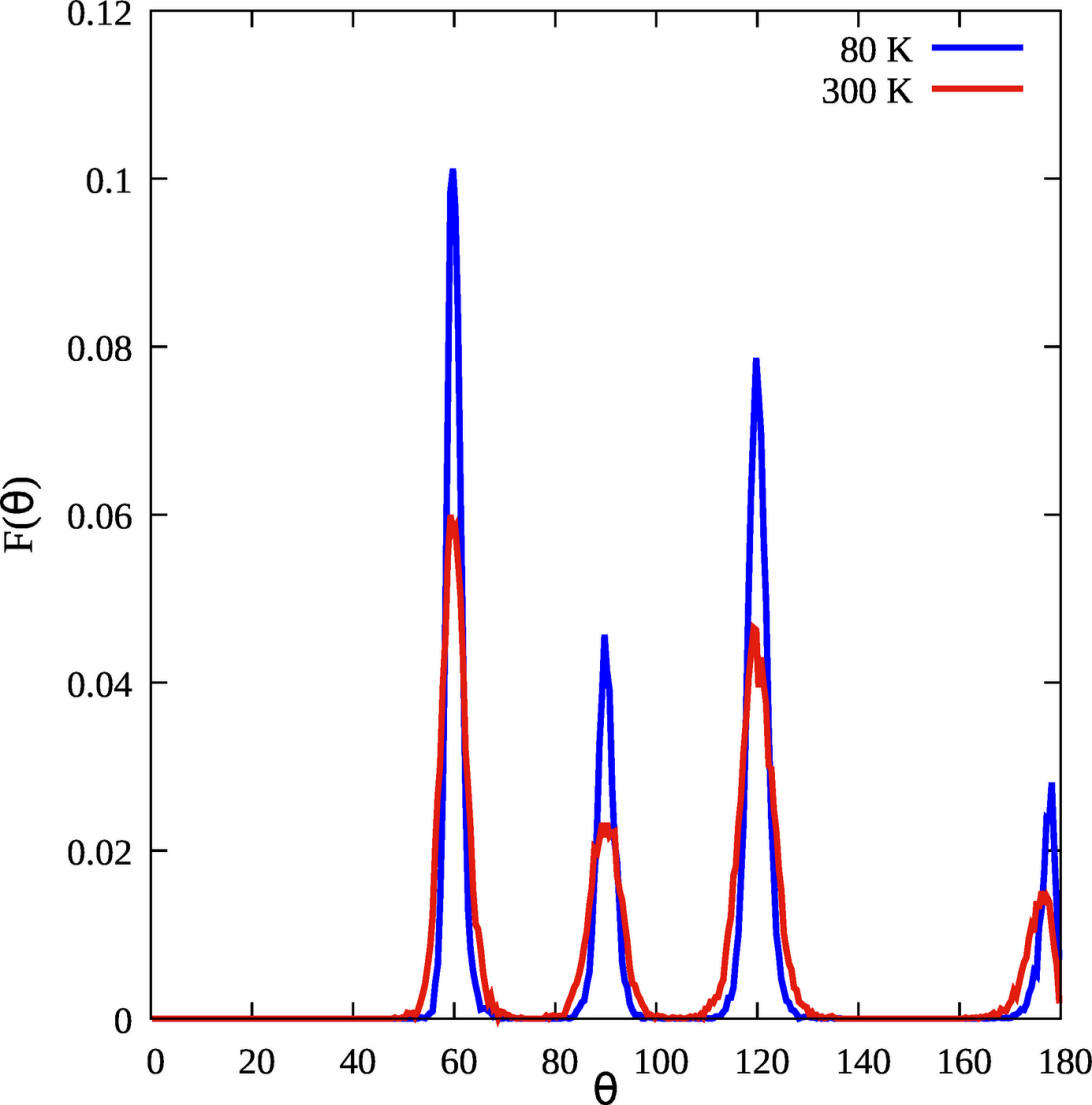
The normalized bond–angle distribution *F*(θ) of solid Au at 80 K and 300 K.

Clearly, the triplet distribution is characteristic of the f.c.c. structure and this can be clearly seen looking at the normalized bond–angle distributions *F*(θ) reported in Fig. 3 ▸ obtained with the same cut-off distance. The peaks at 60, 90, 120 and 180° are the triangular configurations formed by first neighbors and are related to the first four coordination shells. The peaks of the bond–angle distribution are broader at 300 K than at 80 K due to the larger angle variance (see also Tables 1 ▸ and 2 ▸) due to the increase of the thermal disorder. The peak at a mean angle of θ = 180° is an extremal case of three atoms perfectly aligned which is never realized due to the vibrational motion of the atoms. Therefore the angles fluctuate around 180° with a null probability for the perfect alignment so that the distribution [see Filipponi & Di Cicco (1995 ▸) and Di Cicco *et al.* (2018 ▸)] shows a peak at smaller bond angles, with a maximum at θ_M_ = 180 − δ_θ_ where δ_θ_ is related to the variance of the angle distribution {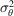 = 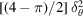}.

### 2.2. Liquid Au at 1400 K

Before performing full RMC analysis including EXAFS data, two stages of RMC refinements were carried out to obtain the initial configuration with 1372 Au atoms in 7 × 7 × 7 unit cells; the first stage was done with 2000 RMC steps without any constraints from EXAFS and XRD (density is the only constraint) to produce a starting configuration with a fully disordered structure. In the second stage, introducing the constraints based on XRD data, the initial configuration was produced after 1000 RMC steps, which provided RMC configurations compatible with XRD data only. Those two steps of the RMC procedure before inserting EXAFS data in the refinement are technically useful to reduce computational time (required RMC steps for convergence) to obtain equilibrium configurations.

In the case of liquids, only two-body contributions γ^(2)^ were taken into account as we verified that three-body and higher-order contributions are small enough to be negligible.

The RMC refinement including EXAFS data was performed using 1000 RMC steps reaching equilibrium after about 100 RMC steps.

**Figure 4:**
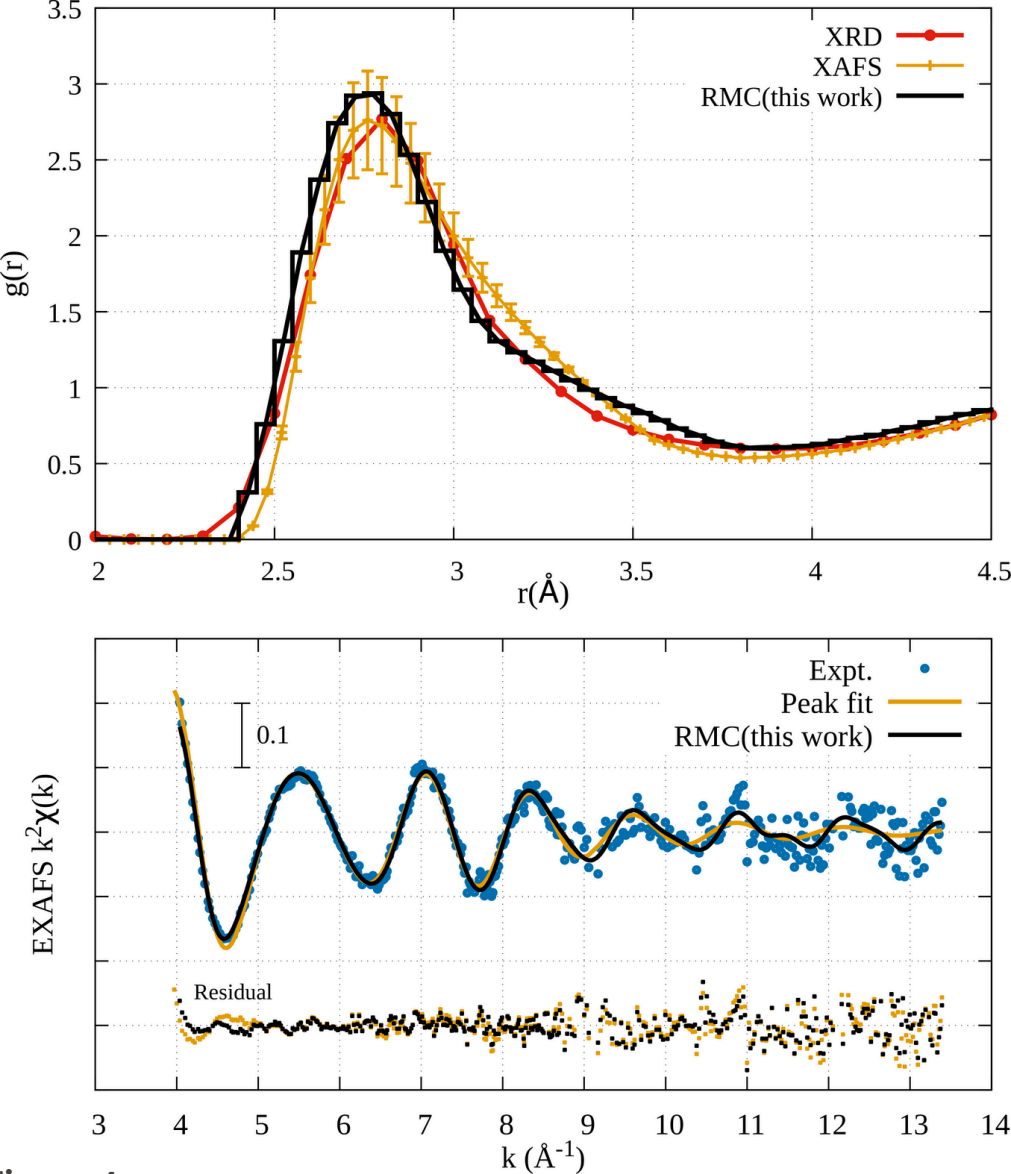
The pair distribution function *g*(*r*) of liquid Au (upper panel) by RMC refinement (black line) is compared with *g*(*r*) based on EXAFS [orange line (Hara *et al.*, 2023 ▸)] and XRD [red line (Waseda, 1980 ▸)]. EXAFS signals of the liquid gold Au *L*_3_-edge (lower panel) by RMC refinement (black line) is compared with experimental data (blue dots) and the analysis by peak fitting [orange line (Hara *et al.*, 2023 ▸)].

The EXAFS data of Au liquid (Fig. 4 ▸, lower panel) used in the RMC refinement were those presented by Hara *et al.* (2023 ▸) and the quality of the RMC refinement was of the same level (similar values of the residual functions) of that published in that publication using the peak-fitting approach. Final results were averaged over the equilibrated configurations and do not depend critically on the choice of the RMC cut-off constraints.

In Fig. 4 ▸ (upper panel), the reconstructed *g*(*r*) by the RMC approach (black curve) shows similar behavior in terms of the position of the peak maximum (around 2.75 Å) as it was obtained previously by XAFS (Hara *et al.*, 2023 ▸) (orange curve) peak-fitting analysis with constraints. They are both shifted to shorter distances with respect to the one based on XRD (red curve). Differences between the previous EXAFS reconstruction and the present one can be seen both at very short distances around 2.5 Å and in the region between 3 and 3.5 Å. The first-neighbor peak results are more clearly defined and sharper in the present *g*(*r*) reconstruction, although the coordination number *N* of first neighbors is equivalent in the models (*N* ≃ 12 for a 3.8 Å cut-off distance).

Differences between the reconstructed *g*(*r*) using the constrained EXAFS peak-fitting technique [see Filipponi (2001 ▸) and Trapananti & Di Cicco (2004 ▸)], where the total coordination number, the area of the decomposed two Γ-like peaks from the first peak of *g*(*r*), and the total second moment are the constraints and the more flexible RMC procedure constrained from the *g*(*r*) itself (and density) are anyway expected as the *g*(*r*) curve does not depend on specific models for the peak shapes.

**Figure 5:**
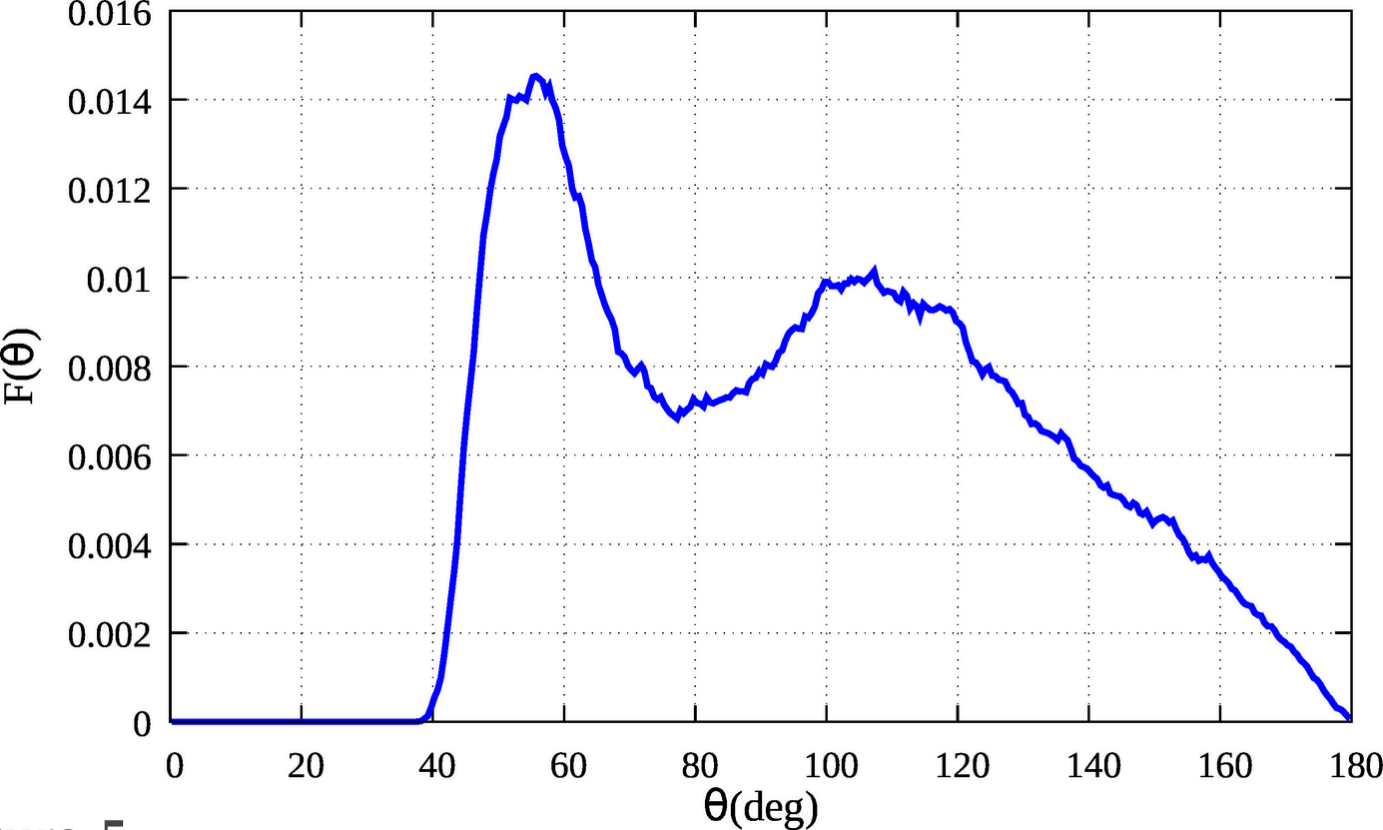
The normalized bond–angle distribution of liquid Au reconstructed by the present RMC refinement.

In order to study the first-neighbor geometrical configurations, we have chosen the first minimum in the pair distribution function, *R*_cut_ = 3.8 Å, as a cutoff distance for bond–angle distributions. As shown in Fig. 5 ▸ the bond angle distribution *F*(θ) based on RMC configurations shows two distinct peaks characteristic of close-packing liquids. A sharper structure with a maximum at about 55° and the other broadened one at around 110° is observed in Fig. 5 ▸. As has been shown in some previous papers of our group (Di Cicco *et al.*, 2003 ▸; Celino *et al.*, 2007 ▸; Di Cicco & Trapananti, 2007 ▸; Celino *et al.*, 2010 ▸; Di Cicco *et al.*, 2014 ▸; Iesari & Cicco, 2016 ▸), the average local structure of close-packing liquids is due to several geometrical arrangements including icosahedral and nearly icosahedral structures in different proportions. In particular, equilateral triangle-like arrangements corresponding to the first peak are consistent with icosahedral structures made up of 20 equilateral triangles. The second peak, which is at approximately 110°, is consistent with the internal angle of a regular pentagon, which is 108°, and can also originate from icosahedra or fragments of icosahedral structures.

**Figure 6:**
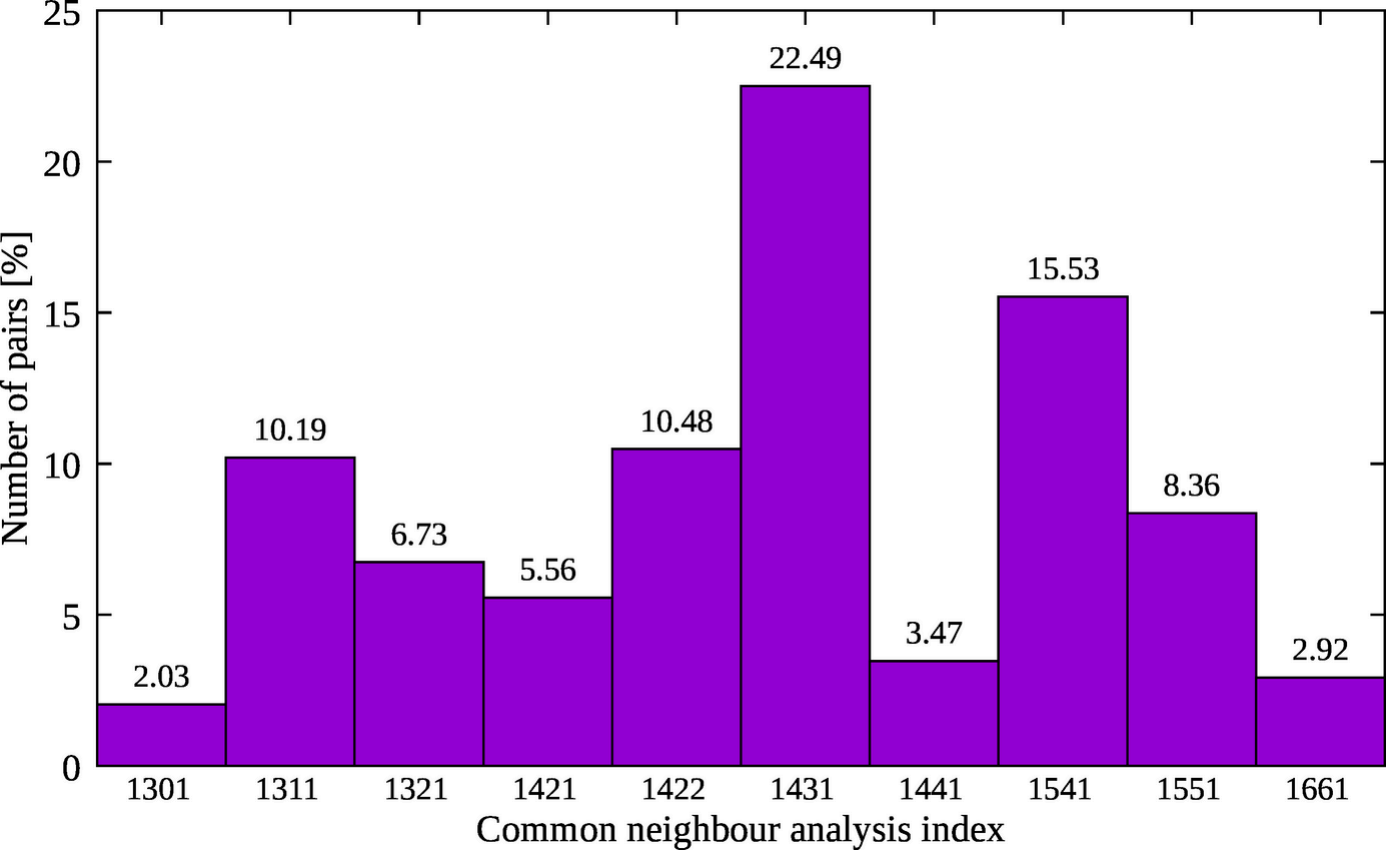
Results of the common neighbor analysis for liquid Au. Only configurations corresponding to more than 2% occurrence are shown.

Investigation of local symmetry configurations has been carried by common-neighbor analysis (CNA) (Honeycutt & Andersen, 1987 ▸; Iesari *et al.*, 2020 ▸), which classifies each pair of nearest-neighbor atoms by a set of four indexes. Within this approach we introduce an index *ijkl*: *i* is the shell number, *j* is the number of nearest neighbors common to both atoms, *k* is the number of bonds between the common neighbors and *l* represents the different bond configurations for given *j*, *k*. In this case, *i* = 1 since only the first peak of *g*(*r*) was considered with the cut-off distance 3.8 Å corresponding to the minimum of the *g*(*r*) after the first peak. As shown in Fig. 6 ▸, the distorted or defective icosahedral (*jk* = 43 and 54 pairs) structures result in being the dominant configurations (22.49% and 15.53%), then hexagonal close-packed (h.c.p.) and f.c.c. (*jkl* = 422 and 421) are 10.48% and 5.56%, respectively. Defective f.c.c.-like structures are possibly associated with *jkl* = 311 (10.19%) and *jkl* = 321 (6.73%) configurations. The 8.36% of the total configuration is a ‘perfect’ icosahedral structure (*jk* = 55) and the others are fragments of different structures.

## 3. Conclusions

We successfully performed local structure analysis of solid and liquid Au using *RMC-GNXAS* providing a combined refinement of Au *L*_3_-edge EXAFS (short-range) and XRD (long-range) data. The analysis confirmed that the EXAFS signals from three-body contributions γ^(3)^ are not negligible in the solid phase using the RMC approach. A new version of the *RMC-GNXAS* program allowing for the direct inclusion of γ^(3)^ contributions was used in this work. As the inclusion of these signals is computationally expensive, we compared the RMC results with those obtained using only two-body γ^(2)^ signals [removing the total simulated three-body 〈γ^(3)^〉 contribution of previous works from the original data]. We then showed that both procedures may be acceptable when the three-body contribution is weak and the main details of the structure are known. The RMC refinements have been then used to derive the best-fit parameters of the first four coordination shells in solid Au at 80 and 300 K. Moreover, we have reconstructed the bond–angle distribution of solid Au and determined the covariance matrix for the first four triplet configurations at 60, 90, 120 and 180°.

The *RMC-GNXAS* procedure was applied to Au EXAFS data in the liquid phase and the pair distribution function *g*(*r*) obtained by RMC refinement was compared with previous results (Hara *et al.*, 2023 ▸; Waseda, 1980 ▸). A slight shift in position of the first-neighbor peak with respect to the XRD determination is confirmed. The first-neighbor distribution results to be slightly sharper than previous determinations. The resulting bond–angle distribution was found to be in line with those of typical close-packing liquid metals and a deeper analysis of the local configurations confirms the occurrence of icosahedral (∼8.4%) and distorted or defective icosahedral (∼38.0%) structures also in this liquid.

These results show that robust and reliable structural results can be obtained by a suitable application of *RMC-GNXAS* to EXAFS data, constraining the long-range structure with complementary data obtained by diffraction or other techniques.

## Figures and Tables

**Table:** Interatomic distances *R*, variances 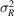, asymmetric parameter (skewness) β and kurtosis τ are listed up to the fourth shell, as well as the average bond angles θ, angle variances 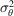, bond–bond ρ_*RR*_ and bond–angle ρ_*R*θ_ correlations. The errors in parenthess are only related to the fluctuations of the parameters for the considered RMC configurations (see text).

80 K	*R*_1_ (Å)	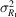 (10^−3^ Å^2^)	β	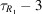
Model	2.876	2.76	0.19	…
RMC (*A*)	2.8733 (4)	2.84 (6)	0.21 (3)	0.20 (23)
RMC (*B*)	2.8705 (3)	2.68 (2)	0.083 (17)	−0.06 (10)

**Table:** 

	*R*_2_ (Å)	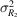 (10^−3^ Å^2^)	β	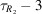
Model	4.068	3.10	0.0000000	…
RMC (*A*)	4.0676 (4)	3.59 (5)	0.099 (58)	−0.09 (12)
RMC (*B*)	4.0689 (3)	4.07 (11)	0.158 (20)	−0.15 (19)

**Table:** 

	*R*_3_ (Å)	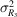 (10^−3^ Å^2^)	β	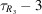
Model	4.983	3.16	0.0000000	…
RMC (*A*)	4.9820 (2)	3.51 (3)	0.0096 (156)	−0.13 (7)
RMC (*B*)	4.9818 (1)	3.64 (7)	−0.072 (19)	−0.15 (17)

**Table:** 

	*R*_4_ (Å)	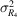 (10^−3^ Å^2^)	β	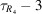
Model	5.754	3.19	0.0000000	…
RMC (*A*)	5.7508 (3)	3.27 (5)	−0.021 (13)	−0.44 (8)
RMC (*B*)	5.7515 (1)	4.38 (4)	0.045 (44)	−0.08 (7)

**Table:** 

Bond angle	θ_1st_ (°)	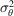 (deg^2^)	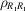	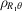
RMC (*A*)	60.048 (5)	2.18 (3)	0.137 (8)	−0.319 (6)
RMC (*B*)	60.095 (4)	2.32 (4)	0.141 (7)	−0.323 (6)

**Table:** 

	θ_2nd_ (°)	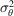 (deg^2^)	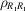	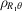
RMC (*A*)	90.053 (4)	3.11 (5)	0.0044 (51)	−0.293 (12)
RMC (*B*)	90.099 (7)	3.42 (5)	0.024 (4)	−0.264 (4)

**Table:** 

	θ_3rd_ (°)	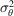 (deg^2^)	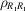	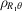
RMC (*A*)	120.028 (5)	3.69 (7)	−0.180 (5)	−0.232 (10)
RMC (*B*)	120.086 (3)	4.09 (5)	−0.157 (6)	−0.229 (2)

**Table:** 

	θ_4th_ (°)	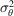 (deg^2^)	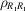	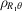
RMC (*A*)	177.44 (2)	2.19 (5)	−0.346 (16)	−0.010 (15)
RMC (*B*)	177.288 (24)	2.50 (8)	−0.267 (13)	0.027 (7)

**Table:** Interatomic distances *R*, variances 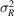, asymmetric parameter (skewness) β and kurtosis τ are listed up to the fourth shell, as well as the average bond angles θ, angle variances 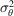, bond–bond ρ_*RR*_ and bond–angle ρ_*R*θ_ correlations. The errors in parentheses are only related to the fluctuations of the parameters for the considered RMC configurations (see text).

300 K	*R*_1_ (Å)	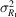 (10^−3^ Å^2^)	β	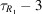
Model	2.886	8.67	0.33	…
RMC (*A*)	2.8864 (1)	9.43 (7)	0.44 (3)	0.15 (9)
RMC (*B*)	2.8864 (3)	9.54 (7)	0.43 (2)	0.14 (5)

**Table:** 

	*R*_2_ (Å)	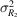 (10^−3^ Å^2^)	β	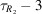
Model	4.081	9.81	0.0000000	…
RMC (*A*)	4.08235 (4)	13.52 (8)	0.073 (13)	−0.04 (7)
RMC (*B*)	4.08290 (2)	14.24 (6)	0.047 (6)	0.05 (3)

**Table:** 

	*R*_3_ (Å)	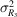 [10^−3^ Å^2^]	β	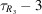
Model	4.999	10.16	0.0000000	…
RMC (*A*)	4.9980 (2)	10.45 (8)	−0.019 (18)	−0.28 (6)
RMC (*B*)	4.9980 (1)	10.80 (6)	0.014 (10)	−0.22 (3)

**Table:** 

	*R*_4_ (Å)	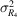 [10^−3^ Å^2^]	β	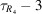
Model	5.772	10.31	0.0000000	…
RMC (*A*)	5.7687 (5)	9.15 (13)	−0.042 (18)	−0.38 (10)
RMC (*B*)	5.7685 (4)	8.59 (10)	−0.049 (16)	−0.26 (7)

**Table:** 

Bond angle	θ_1st_ (°)	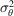 (deg^2^)	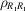	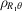
RMC (*A*)	60.025 (4)	6.40 (4)	0.178 (7)	−0.334 (4)
RMC (*B*)	60.019 (6)	6.47 (3)	0.191 (5)	−0.324 (2)

**Table:** 

	θ_2nd_ (°)	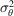 (deg^2^)	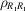	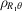
RMC (*A*)	89.978 (8)	9.10 (7)	0.029 (4)	−0.286 (4)
RMC (*B*)	90.002 (7)	9.07 (3)	0.023 (1)	−0.274 (3)

**Table:** 

	θ_3rd_ (°)	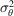 (deg^2^)	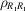	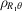
RMC (*A*)	119.941 (6)	10.59 (8)	−0.218 (6)	−0.246 (2)
RMC (*B*)	119.937 (6)	10.62 (5)	−0.217 (4)	−0.249 (2)

**Table:** 

	θ_4th_ (°)	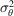 (deg^2^)	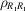	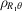
RMC (*A*)	175.64 (2)	4.74 (7)	−0.426 (8)	−0.0074 (36)
RMC (*B*)	175.685 (8)	4.91 (4)	−0.461 (5)	−0.0077 (36)
